# Audit on Prolactin Monitoring for Patients on Oral/Intramuscular Risperidone and Intramuscular Paliperidone

**DOI:** 10.1192/bjo.2025.10684

**Published:** 2025-06-20

**Authors:** Meekha Suresh

**Affiliations:** CWP NHS Trust, Macclesfield, United Kingdom

## Abstract

**Aims:** Risperidone is a commonly used antipsychotic drug in the LD population. One of its common side effects is hyperprolactinemia, which can cause a range of symptoms. Women may experience oligomenorrhoea, amenorrhoea, galactorrhoea (breast milk production), and decreased libido. Men may experience decreased libido, erectile dysfunction, gynaecomastia, infertility, decreased bone mass, and galactorrhoea. These symptoms may go unnoticed in the LD population and lead to behavioural changes.

BNF advises monitoring of prolactin at baseline, after 6 months and then annually.

**Methods:** Identify patients on either oral or intramuscular risperidone and those receiving intramuscular paliperidone within the psychiatry case load. Determine whether these patients have their prolactin levels checked annually. All patients assessed in the East CLDT psychiatry clinic who are on risperidone or paliperidone should have their prolactin levels monitored at least once a year.

**Results:** Out of 106 patients, we identified 27 patients on risperidone (25.4%). 8 out of the 27 patients did not have their prolactin levels checked within the last year (29.6%). 8 out of 19 patients (42.4%) who did have their prolactin levels checked within the last year did not have them checked annually previously. This indicates that approximately 60% of patients are not receiving regular monitoring of their prolactin levels.

**Conclusion:** Approximately 60% of our patients are not having their prolactin levels checked annually. To address this, we can enhance our communication by including a recommendation in our clinic letters to GPs, urging them to include prolactin testing in the annual health check blood work. Additionally, it is essential to regularly monitor symptoms of hyperprolactinaemia during our clinic visits and to educate caregivers about these issues. Symptoms such as gynaecomastia can cause discomfort and may lead to behavioural changes in individuals with intellectual disabilities. By prioritizing these measures, we can improve patient care and outcomes significantly.

